# Genome Sequence of the Thermophilic Cyanobacterium *Thermosynechococcus* sp. Strain NK55a

**DOI:** 10.1128/genomeA.01060-13

**Published:** 2014-01-30

**Authors:** Sergey Stolyar, Zhenfeng Liu, Vera Thiel, Lynn P. Tomsho, Nicolas Pinel, William C. Nelson, Stephen R. Lindemann, Margie F. Romine, Shin Haruta, Stephan C. Schuster, Donald A. Bryant, Jim K. Fredrickson

**Affiliations:** a Pacific Northwest National Laboratory, Richland, Washington, USA; b Department of Biochemistry and Molecular Biology, the Pennsylvania State University, University Park, Pennsylvania, USA; c Institute for Systems Biology, Seattle, Washington, USA; d Department of Biological Sciences, Graduate School of Science and Engineering, Tokyo Metropolitan University, Tokyo, Japan

## Abstract

The genome of the unicellular cyanobacterium *Thermosynechococcus* sp. strain NK55a, isolated from the Nakabusa hot spring, Nagano Prefecture, Japan, comprises a single, circular, 2.5-Mb chromosome. The genome is predicted to contain 2,358 protein-encoding genes, including genes for all typical cyanobacterial photosynthetic and metabolic functions. No genes encoding hydrogenases or nitrogenase were identified.

## GENOME ANNOUNCEMENT

Cyanobacteria, oxygenic phototrophs, thrive in diverse environments—from hot, arid deserts to ice-bound, polar environments and from the surface waters of freshwater lakes or oceans to deeper waters and soils. They occur as free-living, single-celled organisms, but they also provide the foundation for complex communities, some forming symbioses with eukaryotic organisms. Cyanobacteria are found in microbial mats of alkaline hot springs on all continents. These mats represent highly organized communities, in which cyanobacteria are responsible for the majority of carbon fixation. The cyanobacterium *Thermosynechococcus* sp. strain NK55a (NBRC 108920) was isolated from a green microbial mat at the Nakabusa hot spring, Nagano Prefecture, Japan. NK55a and related strains are the major oxygenic photosynthetic organisms in mats growing at moderate temperatures of 52 to 60°C (1). The 16S rRNA gene of NK55a was almost identical (2 mismatches) in sequence to that of *Thermosynechococcus elongatus* BP-1, whose genome was previously described (2).

Purified genomic DNA was sequenced in a 454 pyrosequencer (GS FLX+, Roche) maintained in the Schuster laboratory at the Pennsylvania State University. A total of 229,966 reads averaging 282 bp were generated and then assembled with the Newbler assembler (Roche) into 19 contigs of at least 500 bp. The average read depth was ~26×. Further assembly and gap closing were managed using Sanger sequencing and the phred/phrap/consed package. The genome sequence was autoannotated using RAST (3) (http://rast.nmpdr.org/).

The genome of NK55a consists of a single circular chromosome of ~2,519,964 bp; we were unable to close a single, gene-internal gap that resulted from amplification and assembly problems arising from a repeat-rich region within a hemagglutinin-like gene. The genome of NK55a is slightly smaller than the genome of *T. elongatus* BP-1 (2), displaying 94% average nucleotide identity for coding regions and containing 99 unique genes. Among them are several colocalized open reading frames comprising a sequence of about 8 kb with high homology to *Roseiflexus castenholzii*. Only 1 insertion element was detected in NK55a, while 77 are present in BP-1. Phages or genes associated with phages were not identified in either the NK55a or BP-1 genomes. The genome contains a single rRNA gene cluster.

The core metabolism, photosynthetic apparatus, and cellular processes encoded in the NK55a genome are similar to those found in other cyanobacteria (4, 5). The NK55a genome encodes a type I clustered regularly interspaced short palindromic repeat (CRISPR) immunity system, types I through III restriction-modification systems, and a single toxin-antitoxin system for defense against invasion by foreign DNA. Although hydrogenases commonly occur in cyanobacteria, they are missing in strains BP-1 and NK55a as well as in the type A and B genomes of *Synechococcus* spp., which occur in mat communities associated with hot springs in Yellowstone National Park (6). Unlike the latter strains, NK55a also lacks nitrogenase. Further analysis of this genome and comparative analysis with other genomes will provide further insights into the adaptive mechanisms and evolution of hot spring cyanobacteria.

### Nucleotide sequence accession number.

The genome sequence of *Thermosynechococcus* sp. NK55a is available in GenBank under accession number CP006735.
